# Electron Spin Relaxation of Photoexcited Porphyrin in Water—Glycerol Glass

**DOI:** 10.3390/molecules25112677

**Published:** 2020-06-09

**Authors:** Natalya Sannikova, Ivan Timofeev, Elena Bagryanskaya, Michael Bowman, Matvey Fedin, Olesya Krumkacheva

**Affiliations:** 1 International Tomography Center SB RAS, 630090 Novosibirsk, Russia; sannikova.epr@gmail.com (N.S.); timofeev.itc@gmail.com (I.T.); 2 N.N. Vorozhtsov Institute of Organic Chemistry SB RAS, 630090 Novosibirsk, Russia; 3 Department of Chemistry & Biochemistry, University of Alabama, Tuscaloosa, AL 35487-0336, USA

**Keywords:** spin labels, EPR spectroscopy, porphyrin, electron spin relaxation, distance measurements, DEER/PELDOR

## Abstract

Recently, the photoexcited triplet state of porphyrin was proposed as a promising spin-label for pulsed dipolar electron paramagnetic resonance (EPR). Herein, we report the factors that determine the electron spin echo dephasing of the photoexcited porphyrin in a water–glycerol matrix. The electron spin relaxation of a water-soluble porphyrin was measured by Q-band EPR, and the temperature dependence and the effect of solvent deuteration on the relaxation times were studied. The phase memory relaxation rate (1/T_m_) is noticeably affected by solvent nuclei and is substantially faster in protonated solvents than in deuterated solvents. The T_m_ is as large as 13–17 μs in deuterated solvent, potentially expanding the range of distances available for measurement by dipole spectroscopy with photoexcited porphyrin. The 1/T_m_ depends linearly on the degree of solvent deuteration and can be used to probe the environment of a porphyrin in or near a biopolymer, including the solvent accessibility of porphyrins used in photodynamic therapy. We characterized the noncovalent binding of porphyrin to human serum albumin (HSA) from 1/T_m_ and electron spin echo envelope modulation (ESEEM) and found that porphyrin is quite exposed to solvent on the surface of HSA. The 1/T_m_ and ESEEM are equally effective and provide complementary methods to determine the solvent accessibility of a porphyrin bound to protein or to determine the location of the porphyrin.

## 1. Introduction

Pulsed dipolar electron paramagnetic resonance (PD EPR) is a powerful technique for nanoscale distance measurements in biomolecules, including large, disordered systems [1,2,3,4,5,6]. The dipole–dipole interaction between two spin labels attached to the biomolecule is measured as a periodic modulation of an EPR signal. This interaction scales with the distance, *r*, between the labels as 1/*r*^3^, providing accurate values for the average distance between the labels and even the distribution of distances from the different structural conformations present in the sample. The common spin labels used in PD EPR (most often represented by double electron–electron resonance, DEER or PELDOR [7,8]) are nitroxide radicals because of their small size and the abundance of methods for site-directed spin-labelling (SDSL) of biomolecules [9]. However, the sensitivity of DEER with nitroxide labels is often insufficient, and measurements of long distances with label concentrations less than 5–10 µM are rarely feasible [10,11]. Recently, the photoexcited triplet states of porphyrin [12,13,14,15] and fullerene [16] were demonstrated as spin labels for enhanced sensitivity in PD EPR. A conventional 4-pulse DEER sequence measures the distance between the photoactivated label and a conventional, stable label: the EPR spin echo signal from the photoinduced triplet is observed and the stable radical is pumped. The photoexcited triplet state provides a strong spin echo signal for high-sensitivity, light-induced DEER (LiDEER) measurements because of the strong electron polarization of the triplet spin levels. The initial EPR signal intensity depends on the difference in populations of the two spin levels involved in the observed EPR transition. For stable free radicals, this is determined by the Boltzmann factor. For X-band EPR at temperatures of 77 K and 10 K, the difference in populations is roughly 0.27% and 2.1%, respectively. In contrast, the population difference for a photoexcited triplet state is determined by the intersystem crossing, *isc*, rates which are generally temperature independent. For free-base, tetra-aryl porphyrins, the *isc* rates [17,18] produce polarizations on the order of ±30%.

However, the spin polarization and initial signal intensity are not the only factors determining the sensitivity of DEER [2,6,19]. Accurate measurement of the dipolar interaction requires observation of the dipolar modulation for some time in order to determine its frequency and the corresponding distance. Roughly, a half cycle of dipolar modulation is needed to determine its frequency and the average distance between spin labels. For accurate determination of the distribution of distances, about two to four full cycles are needed. Thus, the dipolar signal must be measured for a time (measurement window) of at least τ_0_ = *m*π*r*^3^/(γ^2^*ħ*) = 0.0192 *m r*^3^ μs, where *r* is in nm and *m* is 0.5–4, the number of dipolar cycles observed. During that measurement window, the initial spin echo signal is attenuated by spin relaxation, a major part being the phase memory decay driven by nuclear spin diffusion in the vicinity of the observed electron spin. This decay typically has the form of a “stretched exponential”

(1) $$I\left(t\right)=I_{0}e^{-{\left(\frac{2t}{T_{m}}\right)}^{n}}$$

where *t* is the length of the measurement, T_m_ is the “phase memory time” and *n* depends on the mechanism of dephasing. Nuclear spin diffusion of solvent protons has *n* > 1 and often approaches 2 [20,21,22,23].

Recent successful studies using triplet porphyrin labels reported rather short values of T_m_ for the triplet. A T_m_ of 1.9 µs at 20 K was reported for the photoexcited triplet state of porphyrin attached to a small helical peptide [12]. More recently, Zn-substituted protoporphyrin IX in neuroglobin had an even shorter T_m_ in the order of 0.52 µs at 20 K, while free-base tetraphenyl porphyrin had a value of 3.06 µs [24]. Such values drastically limit the application of those labels for long-distance DEER measurements because the signal is strongly attenuated with the long measurement window needed for long distances (Figure S1), while the noise is not. In order to recover the same signal to noise ratio by signal averaging, the required number of averages increases as the square of the signal attenuation. Thus, when one of the curves in Figure S1 reaches the bottom of the plot, it is attenuated by a factor of 10^4^ from its original value for short measurement windows and distances. To recover a DEER signal of the same quality as at short distances would require 10^8^ averages and 10^8^ more instrument time—impossible demands. The strong spin polarization of a photoexcited triplet label may offset part of this loss of intensity in long-distance measurements but can provide only incremental increases in the useable distance range. When the T_m_ of the triplet does not allow sufficiently long DEER time traces, alternative approaches can be used: relaxation-induced dipolar modulation enhancement (RIDME) [24] and laser-induced magnetic dipole spectroscopy (LaserIMD) [15,25]. However, the advantages of the strong polarization of triplet states are completely lost in these approaches since the thermally-polarized stable radical is observed. Thus, strategies to increase the T_m_ of photoexcited porphyrin are crucial to broaden the scope of LiDEER.

There are several tactics for increasing the T_m_ of stable free radicals, including controlling: the isotopic content and temperature. Success of these tactics varies considerably for stable radicals; the T_m_ of nitroxides seems to be more easily manipulated than that of trityl radicals [19,26,27,28]. There is much less experimental information about their effect on the T_m_ of photoexcited triplet states. We examine here the extent to which the T_m_ of a photoexcited porphyrin can be manipulated and whether it can approach that of the better current spin labels. That would bring the potentials inherent in photoexcited triplets to long distance DEER measurements. We studied the factors determining the electron spin relaxation of water-soluble photoexcited porphyrin 5,10,15,20-Tetrakis-(*N*-methyl-4-pyridyl)porphine (TMPyP4) in a water glycerol matrix to test routes for optimizing the T_m_ in light-induced PD EPR.

## 2. Results and Discussion

### 2.1. T_m_ in Photoexcited TMPyP4

The T_m_ dephasing of the spin echo can have contributions from several relaxation mechanisms [29]: instantaneous and spectral diffusion [21,22,23,30], dynamic averaging of electron–nuclear coupling to inequivalent nuclei within the molecule [22,31], librations of molecules [32,33,34], and nuclear spin diffusion [6,20,21,22,23]

**Figure 1:**
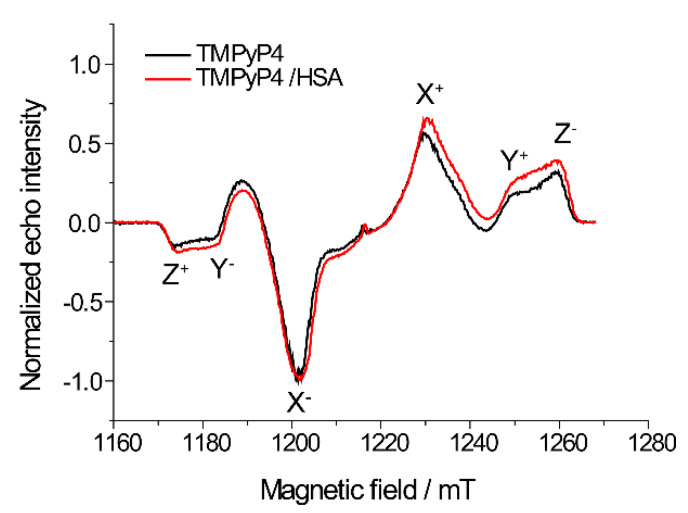
Echo-detected (ED) Q-band EPR spectra of TMPyP4 and TMPyP4/HSA (0.05 mM:0.5 mM) in D_2_O/d_8_-glycerol after laser excitation at 10 K. Z, Y, and X denote canonical transitions where the magnetic field is parallel to the z, y, and x (Scheme 1) axes of the molecule, respectively. Positive amplitudes correspond to absorptive signals and negative ones to emissive signals which are clear indications of strong electron spin polarization.

The nuclear spin diffusion of protons makes the major contribution to T_m_ of nitroxide-based labels with spin **S** = ½ below 50 K [20]. Therefore, to verify the same effect for photoexcited porphyrin with **S** = 1 we have studied the dependence of T_m_ for TMPyP4 on deuteration of the water–glycerol mixture at 10 K, where dynamic processes (molecular motion and conformational flexibility) should be almost damped. The echo decay was studied at 34 GHz (Q-band) in order to reduce the ESEEM arising from hydrogen and the porphyrin core nitrogens. This significantly improved the accuracy of the T_m_ measurements. Upon photoexcitation, strongly polarized EPR spectra (Figure 1) of triplet porphyrin were observed, exhibiting the typical six-line pattern characteristic of a randomly oriented triplet state with strong electron spin polarization and zero-field splitting (ZFS) parameters |D| = 1.27 GHz and |E| = 0.16 GHz (Figure S2). The echo-decay was measured at the magnetic field of the most intense emissive ZFS X^-^ transition (Figure 1).

**Figure 2:**
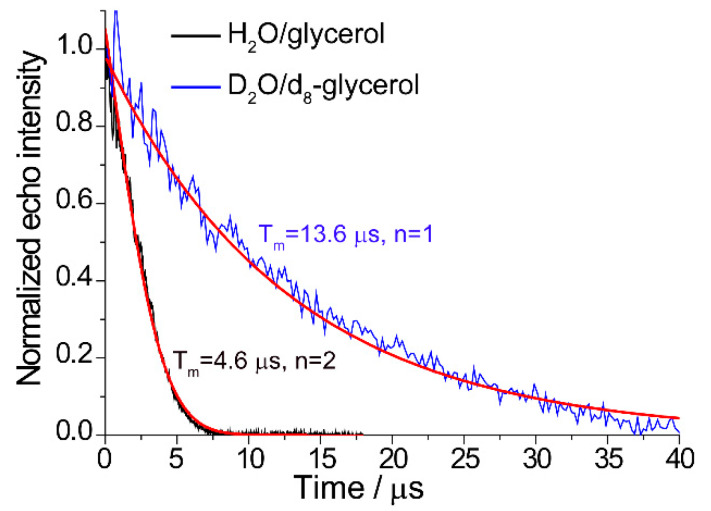
Echo decay for TMPyP4 in different matrices at Q-band at 10 K. Measurements were made at the magnetic field corresponding to the X^−^ canonical orientation of the molecule. Red lines show the best fits by stretched Equation (1) with the parameters indicated.

Deuteration of the solvent often has a strong effect on the shape of the electron spin echo decay of stable free radicals and we observe a similar effect on the photoexcited triplet (Figure 2). The shapes of the echo decays are quite different. The decay in protonated solvent is Gaussian in shape but exponential in the deuterated solvent. In order to make quantitative comparisons, they were fitted by a stretched exponential which is able to fit a wide range of decays including Gaussian and exponential.

In protonated solvent the echo decay is fitted by Equation (1) with T_m_ = 4.6 µs and *n* = 2. Full deuteration of the water–glycerol mixture extends the T_m_ dramatically, giving the value of 13.6 μs and *n* = 1. The magnetic moment of the deuteron is 6.51 times smaller than for the proton, which decreases the effects of the nuclear spins on the electron spin and the rate of spin diffusion among the nuclei. The fact that T_m_ strongly depends on the deuteration of the environment of the porphyrin suggests that the echo signal decay at low temperature is dominated in the protonated glassy matrix by the nuclear spin diffusion due to mutual nuclear spin flip-flops, as it often is for stable free radicals.

**Figure 3:**
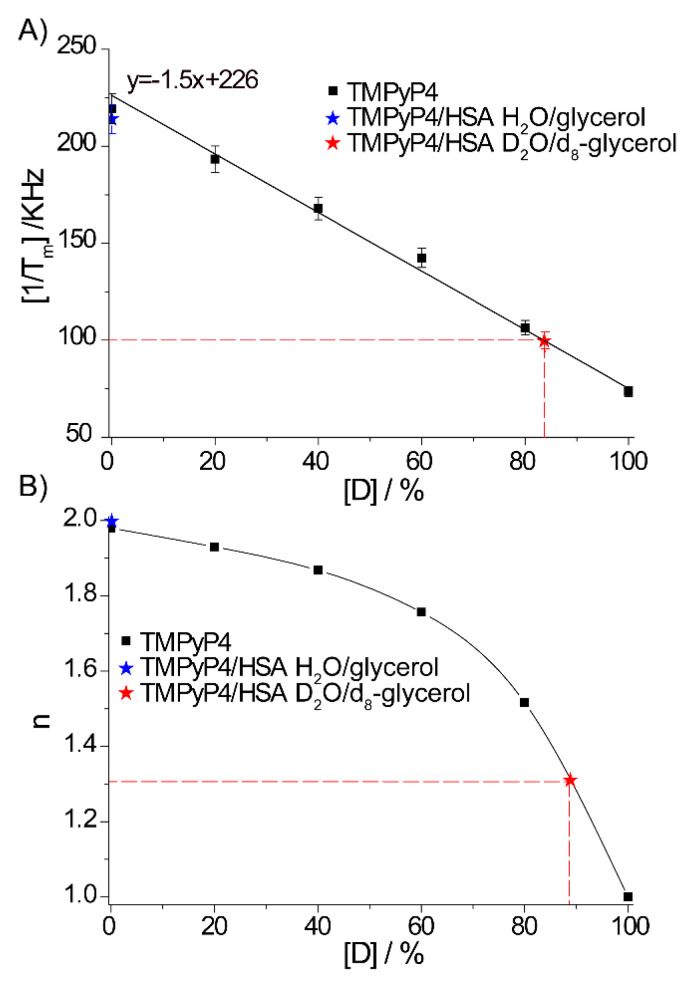
The dependence of phase memory time T_m_ (**A**) and parameter *n* on the percentage of solvent deuteration [D] (**B**). Blue and red stars correspond to values measured for TMPyP4/HSA (0.05 mM:0.5 mM) complex in protonated and deuterated matrices, respectively. The porphyrin/HSA complex in deuterated solvent (red point) has echo decay parameters indicating exposure to solvent over 80–85% of its surface.

When the solvent is only partially deuterated by mixing various amounts of protonated and deuterated solvents, we observed a linear dependence of 1/T_m_ on the degree of solvent deuteration, accompanied by a smooth change in the power *n* of the stretched exponential from 2 to 1 (Figure 3, Figure S3). The decrease of *n* to 1 means that the dephasing due to nuclear spin diffusion becomes so slow in the deuterated environment that other dephasing mechanisms become important.

To gain more understanding of the echo dephasing mechanism in the deuterated surroundings, we tried to measure instantaneous diffusion which limits the T_m_ of most spin labels in solids at all but the lowest concentrations [6]. We examined how the concentration of TMPyP4 and the magnitude of the microwave pulse turning angle, also known as rotation angle or flip angle, impact T_m_ and found that 1/T_m_ is independent of both the porphyrin concentration over the range 0.05–0.25 mM (Figure S4) and the microwave pulse turning angle (Table S1, Figure S5). This result means that, even in the deuterated samples, the phase relaxation has no detectable contribution from spectral diffusion caused by other triplets in the sample, either as instantaneous diffusion or as T_1_ or T_2_-driven spectral diffusion [21,22,23,30].

The T_m_ is slightly orientation dependent: at the Z^−^ peak, T_m_ = 17 µs, which is appreciably longer than the T_m_ = 13.6 µs at the X^−^ orientation (Figure S6). This weak effect is more evident upon comparison of ED spectra measured at different delays between the pulses (Figure S7). A similar dependence of T_m_ on orientation was observed for photoexcited porphycene in toluene and was interpreted in terms of an N–H tautomerism between two equivalent trans structures involving donation of the two N–H protons to the unprotonated nitrogen atoms [35]. In a free-base porphyrin derivative, a dynamic excited-state tautomerization occurred even at 4 K and averaged the |E| value essentially to zero in some cases [36]. Accordingly, fluctuations of |E| produced by tautomerization in triplet state set an upper limit to the T_m_ in the deuterated environment. The fact that |E| does not decrease due to averaging at the studied temperatures, indicates that the rate of interconversion is slow relative to the ZFS parameter |E|; therefore, dephasing in deuterated glass is relatively slow.

**Figure 4:**
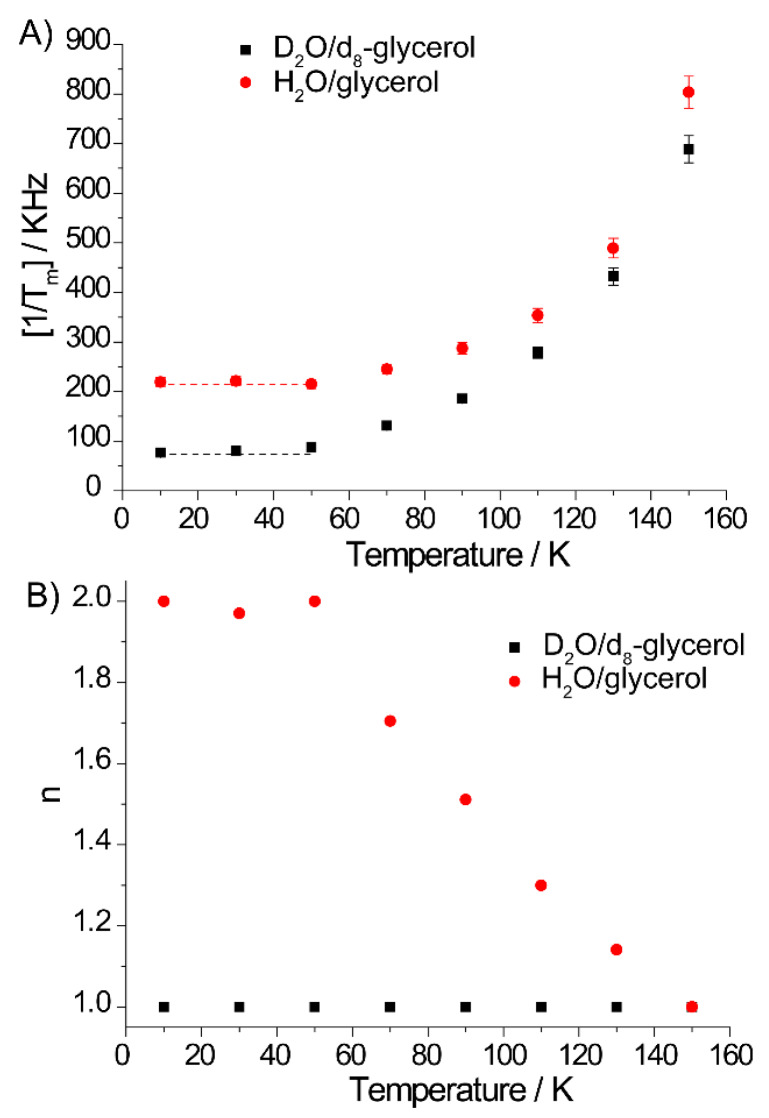
The temperature dependence of T_m_ (**A**) and n (**B**) for TMPyP4 in different matrices.

To understand the underlying mechanisms of T_m_ in more detail, we measured the temperature dependence of T_m_. (Figure 4 and Figure S8). Below 50 K, 1/T_m_ is practically independent of temperature in both protonated and deuterated environments. The parameter *n* remains equal to 2 up to 50 K in protonated glasses, supporting proton spin diffusion as the dominant spin echo dephasing mechanism. Only above 50 K does the 1/T_m_ increase in both protonated and deuterated samples as thermally activated processes become important. Since 1/T_1_ is substantially smaller than 1/T_m_ throughout the studied temperature range (Figure S9), the temperature dependence of 1/T_m_ above 50 K is attributed predominantly to increasing low-amplitude motion modulating the large ZFS.

### 2.2. The Use of T_m_ to Determine Porphyrin Location in a Biopolymer

Porphyrin derivatives are widely used in photodynamic therapy (PDT) of cancer [37]. The important consideration in the development of new drugs for PDT is the interaction of porphyrin with biopolymers, which can act as a delivery system or as a treatment target. The formation of porphyrin-biomolecule complexes with DNA or protein can alter the photophysical properties and efficiency of a PDT agent, depending on its location inside the complex and on its availability to water molecules [38]. We believe that the dependence of 1/T_m_ of a triplet porphyrin on the deuterium content in its surroundings can provide valuable information on the solvent availability of the porphyrin and on its position relative to the surface of the non-covalent complex, just as the 1/T_m_ of a nitroxide spin label covalently bound to a histone component quantitatively responds to deuterated protein around it [26,27].

We used a model complex of TMPyP4 with human serum albumin (HSA) to demonstrate the feasibility of this approach. TMPyP4 is a potential photosensitizer for PDT and is a G-quadruplex stabilizer for telomerase-based cancer therapeutics [39]. HSA is the most abundant protein in blood plasma with multiple binding sites. TMPyP4 forms complexes with HSA with a relatively high binding constant of about K = 10^4^ M^−1^ [40], meaning that about 80% of the porphyrin is bound at the concentrations used of HSA (0.5 mM) and TMPyP4 (0.05 mM). The observed photoexcited ED spectrum for TMPyP4/HSA coincides with that of the unbound porphyrin (Figure 1).

The amounts of protein and solvent protons around the porphyrin in TMPyP4/HSA complexes were determined in natural abundance in glycerol/water buffer and in glycerol-d_8_/D_2_O buffer. T_m_ values for TMPyP4/HSA in the deuterated buffer are quite longer than in the protonated buffer (Figure 3 and Figure S11), indicating that solvent protons play significant roles in the dephasing in the complex with protein. According to calibration curves (Figure 3), the T_m_ = 9.45 µs and the *n* = 1.3 for TMPyP4/HSA correspond to the values observed when the fraction of deuterium surrounding the porphyrin is 80–85%. Such a large amount of deuterium near the porphyrin means that TMPyP4 forms a complex with the surface of the protein and has a high accessibility to solvent molecules, and accordingly, to molecular oxygen, which plays a crucial role in PDT.

To independently verify these conclusions about the location of TMPyP4 obtained from the T_m_ measurements, we investigated the photoexcited triplet state of TMPyP4 using a more standard ESEEM approach [41,42]. Weak hyperfine and quadrupolar couplings between unpaired electron(s) and surrounding nuclear spins produce the ESEEM. The intensity of the ESEEM has been used since the late 1970s for titration and quantitative measurement of numbers of specific isotopes of, e.g., H, C, N, and O, near paramagnetic centers in soluble and membrane proteins, photosynthetic complexes from bacteria and green plants, membranes, materials, and nanoparticle surfaces; and is in use to measure ^2^H solvent nuclei near spin-labeled membrane-associated proteins [43,44,45,46,47,48] and spin-labeled supramolecular complexes [49,50]. We apply it here to quantitate the solvent protons near the photoexcited triplet in its complex with HSA.

**Figure 5:**
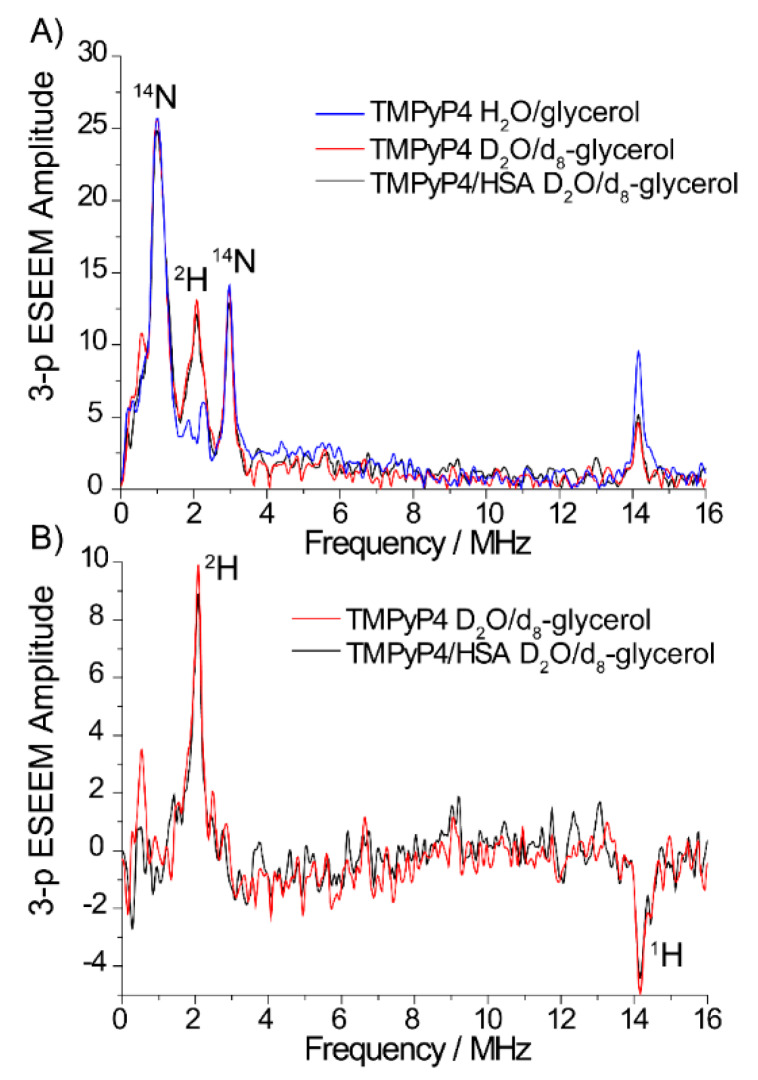
(**A**) Frequency-domain ESEEM spectra of TMPyP4 in water/glycerol matrices (red and blue lines) and in complex with HSA (0.05 mM:0.5 mM of TMPyP4:HSA) in deuterated environment (black lines). (**B**) Frequency-domain ESEEM spectra after subtraction of the spectra in protonated matrices.

**Figure 6:**
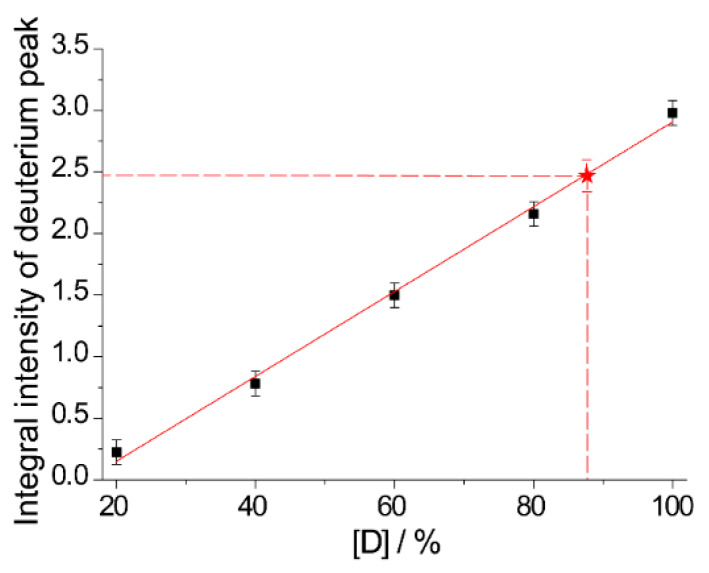
Dependence of the integrated intensity of the deuterium peak on the percentage of solvent deuteration [D]. The porphyrin/HSA complex in deuterated solvent (red point) has an integrated intensity corresponding to exposure to solvent over 85–90% of its surface.

ESEEM spectra (Figure S12) of TMPyP4 were recorded at the magnetic field of the X^−^ line of the triplet state at X-band. Frequency-domain ESEEM spectra (Figure 5 and Figure S13) of photoexcited porphyrin in a protonated glassy solution exhibit strong peaks at 1 MHz and 2.9 MHz from nuclei of nitrogens [51], and also a peak near 14 MHz from protons of TMPyP4 and the solvent located within a nanometer from the porphyrin. When ESEEM experiments are carried out in deuterated matrix, an intense deuterium peak appears at 2.1 MHz, accompanied by a decrease in the proton peak as solvent protons adjacent to the porphyrins are replaced by deuterons. The frequency spectrum in the fully protonated solvent was subtracted from all the spectra to remove the nitrogen peaks and allow precise measurement of the integral intensity of the deuterium peak (Figure 5B). There is a strong linear relation between the integral intensity of the deuterium peak in the ESEEM spectra and the degree of solvent deuteration (Figure 6 and Figure S13). This titration of the deuterium peak integral provides quantitative independent corroboration of the exposure of porphyrin to solvent molecules obtained from T_m_ measurements.

The ESEEM spectra of TMPyP4/HSA immersed in deuterated surroundings reflect the accessibility of the porphyrin to the solvent molecules. The integral intensity of the deuterium peak in the complex of photoexcited TMPyP4/HSA complex is only 85–90% of that for the unbound TMPyP4 in ^2^H solvent (Figure 6). This value coincides well with solvent content near TMPyP4 in complex with HSA estimated by T_m_ measurements. Thus, both approaches, T_m_ and ESEEM, agree on the environment of the porphyrin on the surface of the protein. We obtained very similar T_m_ (Figure S11) and ESEEM results (Figure S14) for TMPyP4 in complex with HSA at two ratios of porphyrin and protein (0.05:0.5 mM and 0.25:0.5 mM). This indicates that at the HSA concentration used almost all TMPyP4 molecules form complexes with HSA and do not form aggregates, in good agreement with the previously published binding constant (K = 10^−4^ M) [40]. The agreement between measurements at the different concentrations of TMPyP4 also shows that their +4 charge does not affect their spatial distribution or relaxation, exactly as predicted by the textbook Debye–Huckel equations at the ionic strength of our buffered protein solutions.

The most exciting result of this work is the good agreement between the degree of deuterium in the surroundings of the triplet that we obtained from the T_m_ and the ESEEM techniques. Said agreement indicates that the ^1^H on the protein is responsible for the increment in the phase memory relaxation in the complex with protein. Consequently, deuteration of the protein would produce essentially the same long T_m_ as for the model system of TMPyP4 in deuterated water/glycerol. Deuteration of the protein seems to offer the same benefits for a photoexcited porphyrin label as it did for histones spin-labelled with a nitroxide [27].

**Figure 7:**
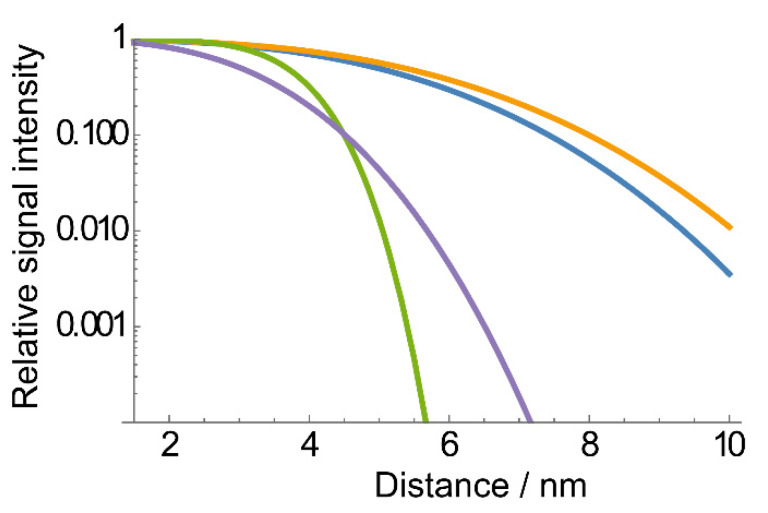
The semilog plot of the calculated attenuation of the initial signal intensity as the minimum measurement window was increased to accommodate two dipolar oscillations for distance measurement. The three curves were calculated from the T_m_ values we measured for excited TMPyP4 triplet states (green curve): 4.6 µs with *n* = 2 for ^1^H water:glycerol; (blue) 13.6 µs with *n* = 1 at the X^−^ peak for ^2^H water:glycerol; and (orange): 17 µs with *n* = 1 at the Z^−^ peak for ^2^H water:glycerol; (violet) the curve for the best previously reported triplet: 3.06 µs and *n* = 1 [24].

When we examine the attenuation of the signal in the measurement window appropriate for DEER, we find that substantially larger distances should become experimentally available with TMPyP4 in a deuterated sample than for previously reported triplet labels, Figure 7.

## 3. Materials and Methods

HSA (A3782) and 5,10,15,20-Tetrakis(1-methyl-4-pyridinio) porphyrin tetra(p-toluenesulfonate) (TMPyP4, 323497) was purchased from Sigma–Aldrich Chem. To prepare the samples, TMPyP4 was dissolved in the protonated or deuterated water–glycerol mixture (1:1) at a 250 μM concentration, and 10 μL volumes were placed into quartz tubes (OD 1.7 mm, ID 1.1 mm) for EPR measurements. In the study of the dependences on the deuteration fraction, the protonated and deuterated stock solutions of TMPyP4 in water–glycerol were mixed in the desired ratios. To prepare the complex of TMPyP4 with HSA at ratio 0.1:1 or 0.5:1 (TMPyP4:HSA), 1 mM of HSA was mixed with 0.1(0.5) mM TMPyP4 in 10 mM PBS (KH_2_PO_4_ and K_2_HPO_4_ in H_2_0 or D_2_0, pH 7.4) solution and was vortexed vigorously. The final concentrations were 0.05 (0.25) mM and 0.5 mM for TMPyP4 and HSA, respectively, after adding 50% of glycerol.

The samples were characterized by optical spectroscopy (Figure S15) employing an Agilent Cary 60 UV-Vis Spectrophotometer. Built-in baseline correction was performed on an empty cuvette; spectra of the samples and solvent were collected with 1 nm steps and 500 ms collection time. In the end, the spectrum of solvent was subtracted from the spectra of the studied samples.

Pulsed EPR data were obtained at X-band and Q-band using a Bruker Elexsys E580 pulse EPR spectrometer equipped with an ER 4118X-MD5 and EN5107D2 resonators and Oxford Instruments temperature control system. The samples were placed into quartz tubes (OD 1.7 mm, ID 1.1 mm) in 10 μL volume for Q-band and into quartz tubes (OD 2.8 mm, ID 1.8 mm) in 40 μL volume for X-band EPR measurements. The measurements were performed over a 10–150 K temperature range. Samples were shock-frozen in liquid nitrogen before placing in the precooled resonator. Samples were photoexcited with the second harmonic of a pulsed Nd:YAG Lotis TII laser (532 nm) with a 1–10 Hz repetition rate. The quantum yield of triplet formation for TMPyP4 is 0.85 [52]. Laser pulses were transmitted to a sample by an optical fiber; the average power measured at the terminus was 5–10 mJ per pulse, enough to excite all TMPyP4 molecules in the sample, which was confirmed since the triplet EPR did not increase if the light power was increased. Laser pulses were 60–70 ns FWHM with 10–20 ns jitter relative to the microwave pulses.

Echo-detected (ED) spectra were collected at Q-band using a standard Hahn echo sequence (laser flash-DAF-π/2-τ-π-τ-echo) with 40 ns π-pulse, 1000 ns delay after flash (DAF), and 300 ns initial τ-delay. T_m_ was measured using a two-pulse Hahn echo; microwave power was varied to test for instantaneous diffusion. T_1_ was measured using the inversion−recovery technique with a π inversion pulse and detection by a two-pulse Hahn echo sequence.

ESEEM time traces were collected at X-band at 10 K using a 3-pulse sequence (laser flash-DAF-π/2-τ-π/2-T-π/2-τ-echo) with a four-step phase cycle, 8 ns π/2-pulse, 1000 ns DAF, 260 ns τ-delay, and 200 ns, initial T value was incremented every ΔT = 8 ns. A detailed description of the processing of ESEEM data is provided in SI.

## 4. Conclusions

In this work, we investigated the factors determining echo dephasing of photoexcited porphyrin in a water glycerol matrix. 1/T_1_ is substantially smaller than 1/T_m_ at temperatures below 100 K and does not contribute to spin-echo decay. The T_m_ is strongly affected by solvent nuclei, and echo dephasing is substantially faster in protonated solvents than in deuterated solvents. T_m_ increases up to 13–17 μs in a deuterated environment; thus, the use of perdeuterated proteins and deuterated matrix potentially can significantly increase the distances available for LiDEER with a photoexcited porphyrin label [9], as previously observed for DEER with nitroxide radicals [26,27,28]. The 1/T_m_ is independent of temperature in both protonated and deuterated matrices below 50 K and significantly increases above 50 K, probably due to increasing low-amplitude motion modulating the large ZFS. There seems to be no need for temperatures below 50 K with its additional liquid helium consumption.

The 1/T_m_ depends on the degree of solvent deuteration and can be used to characterize the environment of a porphyrin in complexes with biopolymers, including those used in photodynamic treatment. Both 1/T_m_ and the intensity of the deuterium ESEEM peak in a porphyrin/protein complex immersed in deuterated surroundings reflect the accessibility of the photoactivated label to the solvent molecules with good accuracy. Therefore, these approaches can be used individually or as complementary methods to characterize the locations of porphyrins.

## Figures and Tables

**Scheme 1:**
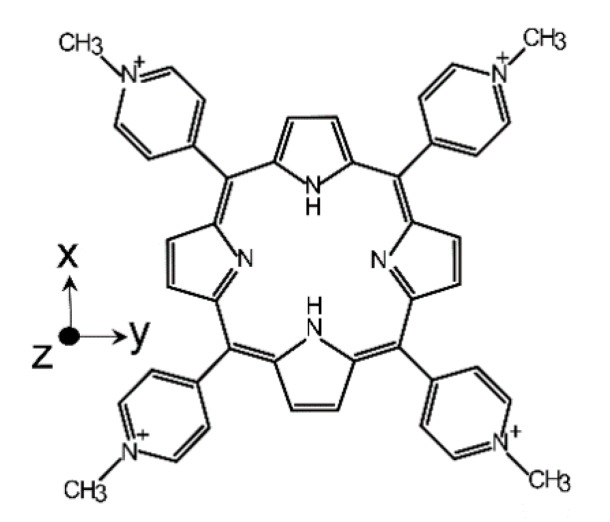
Structure of TMPyP4 and the x,y,z directions of ZFS axes.

## Supplementary Materials

The following are available online. Simulation of ED spectra of TMPyP4; dependence of T_m_ on excitation pulse power; echo decay at different canonical orientations and ED spectra at different delays; T_1_ measurements, echo decay and ESEEM measurements at different ratio of TMPyP4 and HSA; UV-Vis spectra; processing of ESEEM data, all raw data.
