# Supplementary material for: Electron Spin Relaxation of Photoexcited Porphyrin in Water—Glycerol Glass
Source: Molecules. 2020 Jun 9;25(11):2677. doi: 10.3390/molecules25112677 (PMC7321249; doi:10.3390/molecules25112677)
Supplement: Supplementary file 1 [file molecules-25-02677-s001.pdf]

SUPPORTING INFORMATION

Electron Spin Relaxation of Photoexcited Porphyrin in Water-Glycerol Glass

Natalya E. Sannikova,[1] Ivan O. Timofeev,[1] Elena G. Bagryanskaya,[2,*] Michael K. Bowman [2,3*], Matvey V. Fedin[1*] and Olesya A. Krumkacheva[1*]

[1] International Tomography Center SB RAS 630090 Novosibirsk, Russia; sannikova.epr@gmail.com (N.E.S), ivan.timofeev@tomo.nsc.ru (I.O.T)

[2] N.N. Vorozhtsov Institute of Organic Chemistry SB RAS 630090 Novosibirsk, Russia;

[3] Department of Chemistry & Biochemistry, University of Alabama, Tuscaloosa, Alabama 35487-0336, United States;

* Correspondence: olesya@tomo.nsc.ru (O.A.K.), mfedin@tomo.nsc.ru (M.V.F.), mkbowman@ua.edu (M.K.B), egbagryanskaya@nioch.nsc.ru (E.G.B.)

Table of contents

Simulation of ED spectra of TMPyP4 2

Dependence of T_m on excitation pulse power 2

Echo decay at different canonical orientations and ED spectra at different delays 3

T_1 measurements 4

Echo decay and ESEEM measurements at different ratio of TMPyP4 and HSA 5

UV-Vis spectra 6

Processing of ESEEM data 6

Simulation of ED spectra of TMPyP4

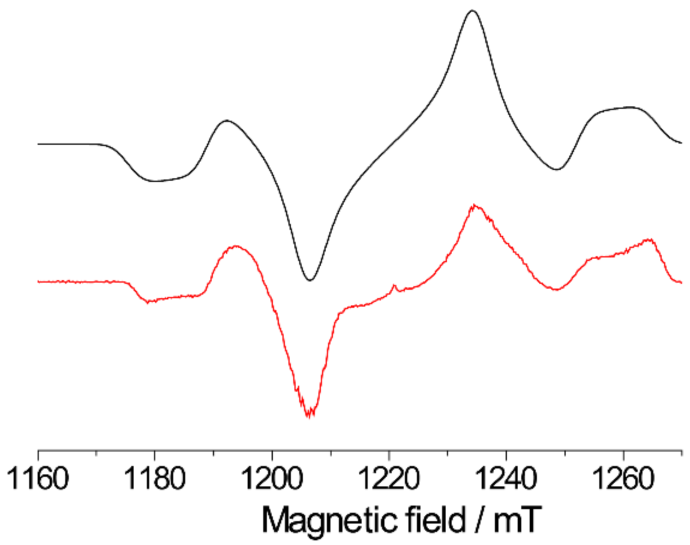

Fig. S1. The experimental (black) and simulated (red) Q-band ED EPR spectra of TMPyP4 in D_2O/d_8-glycerol after laser excitation at 10 K. The spectrum was simulated using Easyspin software by function 'pepper' with zero-field splitting (ZFS) parameters |D|=1.27 GHz and |E|=0.16 GHz.

Dependence of T_m on excitation pulse power

Table S1. Dependence of T_m on turning angle of the microwave pulses. The pulse lengths in 2-p echo sequence were 20 and 40 ns and were attenuated to give 90^0 and 180^0 pulses at 13 dB. Length of pulses was the same in all measurements. The T_m is independent of the achievable level of excitation of the sample as shown by the lack of change in T_m vs. excitation pulse power

Excitation pulse power	13dB	16 dB	19 dB	22dB	25 dB
Tm / μs	13.6	13.7	13.8	13.4	12.4

Echo decay at different canonical orientations and ED spectra at different delays

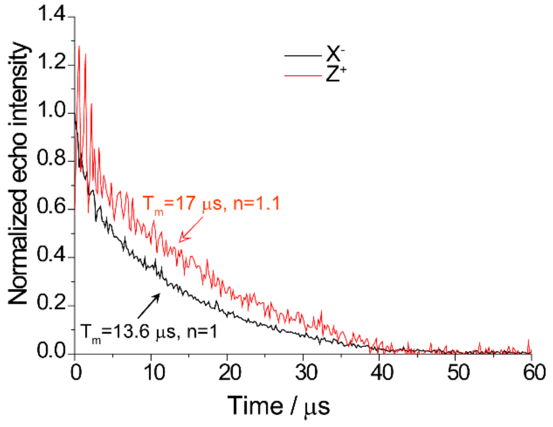

Fig.S2 Echo decay for TMPyP4 at different canonical transitions in fully deuterated matrices at Q-band at 10 K.

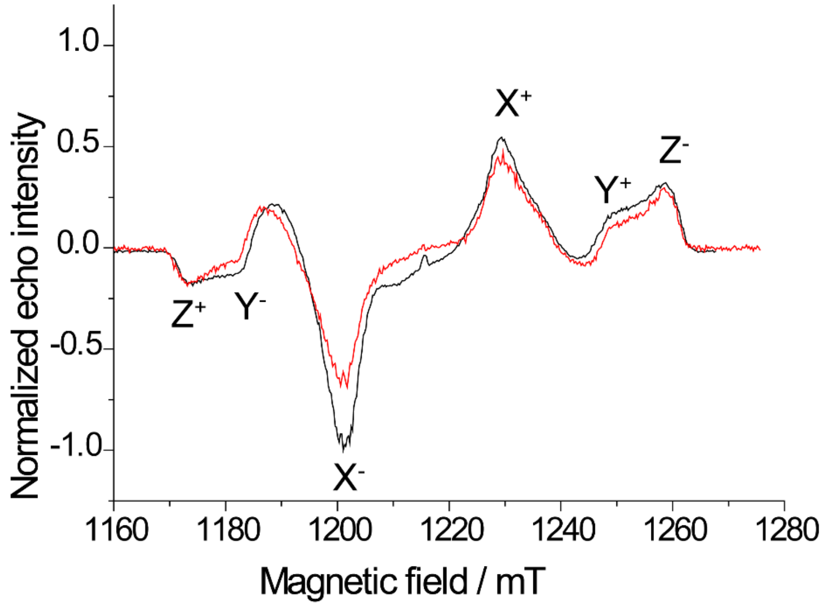

Fig. S3 Echo-detected Q-band EPR spectra of TMPyP4 in D_2O/d_8-glycerol at different delay between MW pulses: black line – 300 ns, red line – 7000 ns

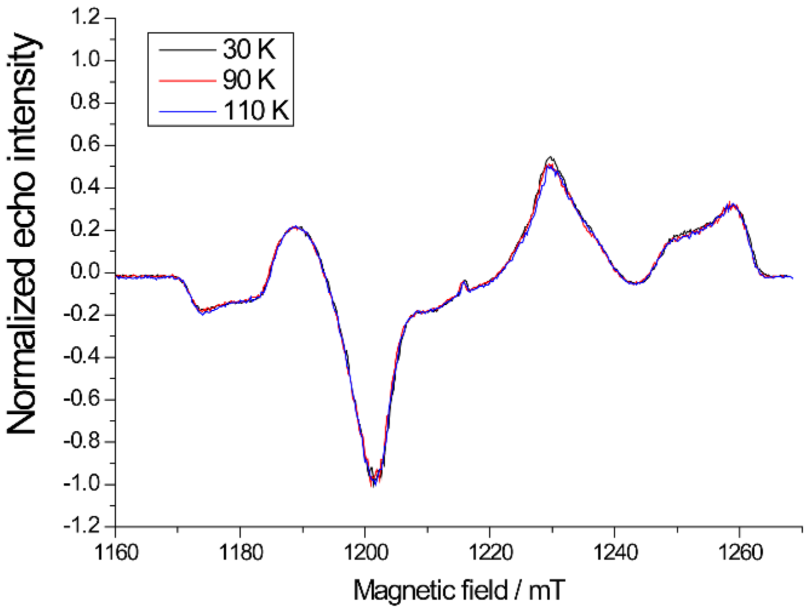

Fig. S4 Echo-detected Q-band EPR spectra of TMPyP4 in D_2O/d_8-glycerol at different temperatures

T_1 measurements

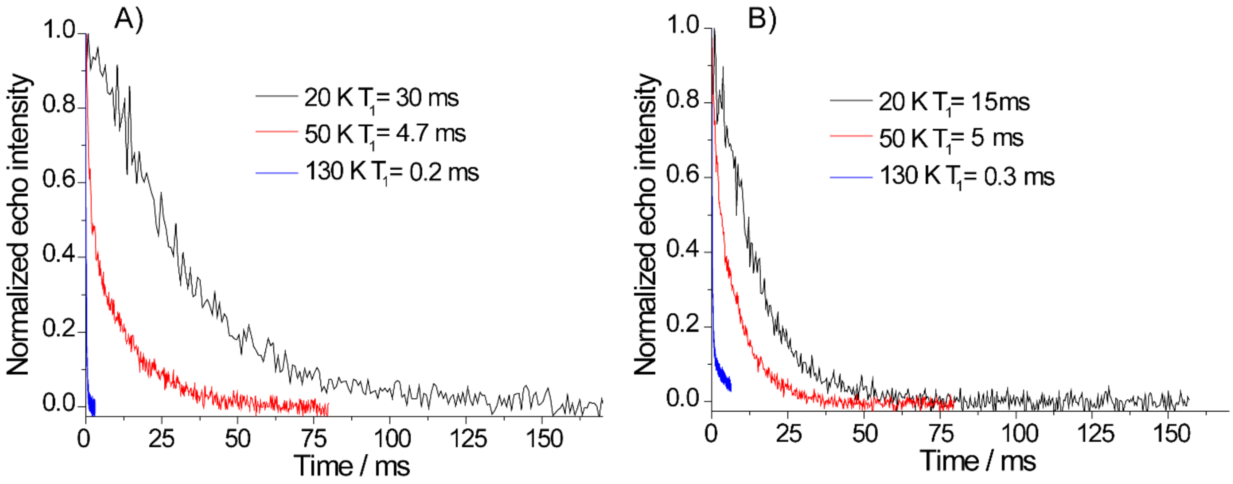

Fig. S5 T_1 measurements at different temperature in A) deuterated and B) protonated matrices. T_m values were estimated as the time during which the spin echo signal decreases by 2.7

Echo decay and ESEEM measurements at different ratio of TMPyP4 and HSA

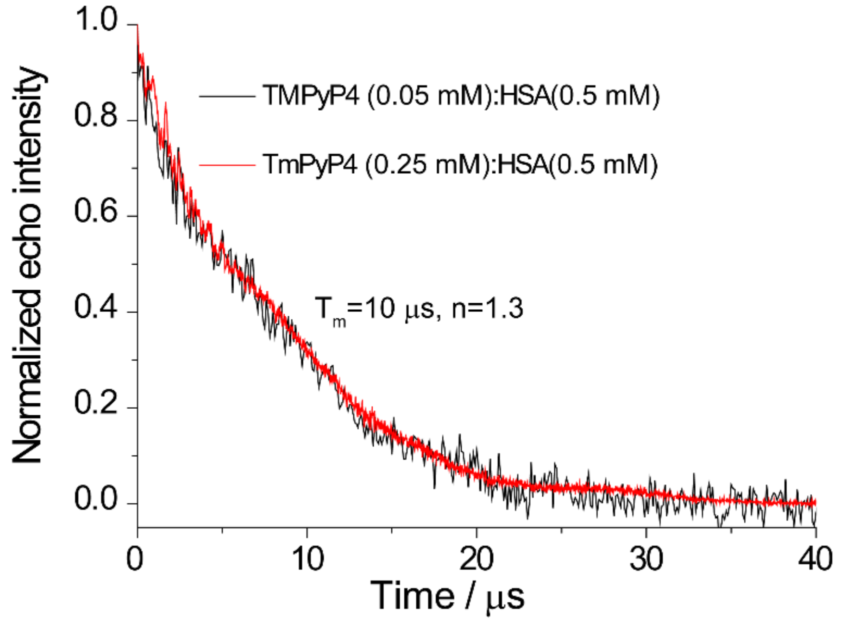

Fig. S6 Echo decay at different ratios of TMPyP4 and HSA (0.05:0.5 and 0.25:0.5) in deuterated matrices at 10K at Q-band.

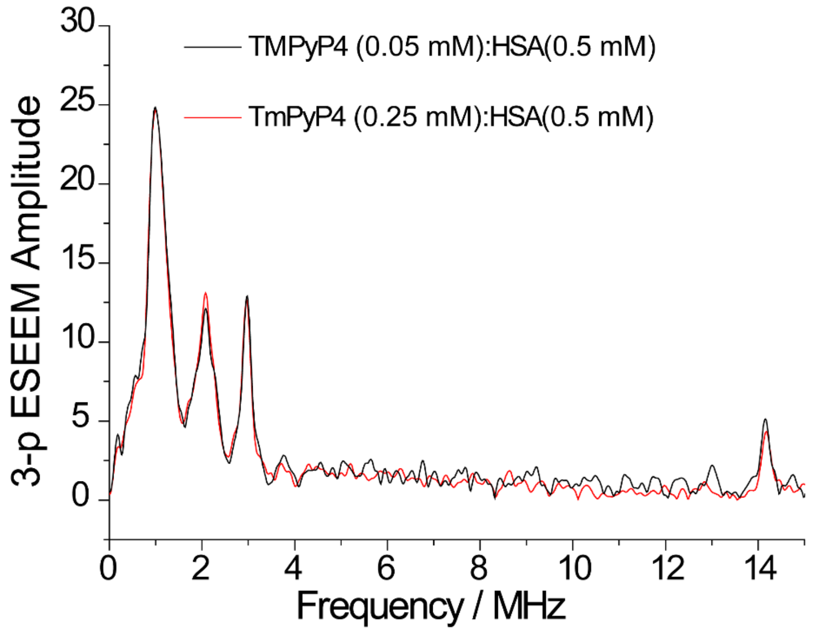

Fig. S7 ESEEM at different ratios of TMPyP4 and HSA (0.05:0.5 and 0.25:0.5) in deuterated matrices at 10 K

UV-Vis spectra

The samples were characterized by optical spectroscopy (Figure S8) employing an Agilent Cary 60 UV-Vis Spectrophotometer. Built-in baseline correction was performed on empty cuvette, spectra of the samples were collected with 1 nm step, 500 ms collection time.

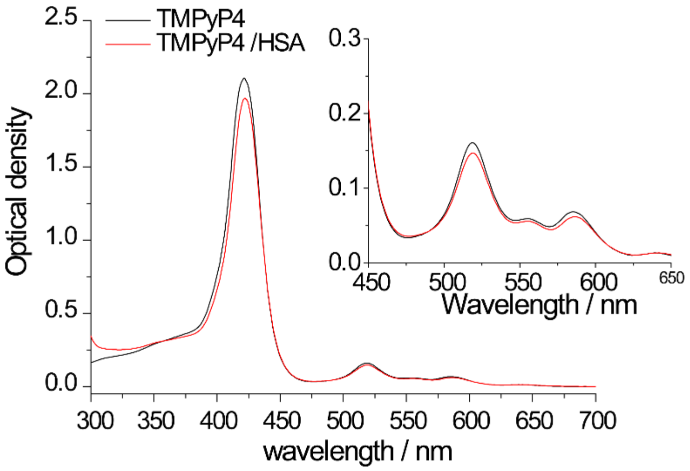

Fig. S8 UV-Vis spectra of 0.5 mM TMPyP4 in buffer (black line) and 0.5 TMPyP4 mM in complex with 1mM HSA (red line), optical path length - 1 mm.

Processing of ESEEM data

The experimental ESEEM data were processed following the algorithm:

1. The real part and logarithm of signal were taken.

2. The time traces were baseline corrected with a second-order polynomial.

3. The time traces were apodised with the same Hamming window (N=8000) and zero-filled to 8096 data points.

4. The magnitude frequency spectrum was calculated using FFT.

5. The frequency spectrum in a fully protonated solvent was subtracted from all spectra to reduce nitrogen peaks and to reveal changes in the proton lines.

6. The integral of the deuterium peak was calculated.

There are several features in this algorithm. First, the logarithm of the ordinate followed by baseline correction allows one to quantitatively compare the Fourier spectra of different samples.

In general, 3-pulse ESEEM modulation for weakly coupled nuclear can be represented as

$$I(T) = AD(T)\prod_i (1 - k_i(1 - \cos w_i\, T))$$

where A is the amplitude of the signal, $D(T)$ is the decay function due to relaxation, the terms $k_i \cos \omega_i T$ account for all modulation contributions.

Then

$$\ln\left(I(T)\right) = \ln A + \ln D(T) + \ln \prod_i (1 - k_i(1 - \cos w_i T))$$

$$= \ln A + \ln D(T) + \sum_i \ln\left(1 - k_i(1 - cos\ w_i T)\right)$$

Since ln(1+x)≈x for small x, and typically $k_i \ll 1$, we can rewrite the last equation as:

$$\ln\left(I(T)\right) = \ln A + \ln D(T) - \sum_i k_i(1 - cos\ w_i T) =$$

$$= \ln A + \ln D(T) - \sum_i k_i + \sum_i k_i cos\ w_i T$$

The polynomial baseline correction removes the first three terms in this expression, and only the third term accounting for modulations will contribute.

$$\ln\left(I(T)\right)_{corr} \approx \sum_i k_i \cos w_i T$$

At the same time, the expression for $I(T)$ can be modified to a similar form:

$$I(T) = AD(T) \prod_i (1 - k_i(1 - \cos w_i T)) \approx AD(T)(1 - \sum_i k_i(1 - \cos w_i T))$$

$$\approx AD(T)(1 + const + \sum_i k_i \cos w_i T)$$

Therefore, Fourier transform of the functions $I(T)$ and $\ln\left(I(T)\right)_{corr}$ should result in similar ratios of ESEEM peaks in frequency-domain spectra, which was additionally verified by us
